# Effects of unconditional cash transfers on the outcome of treatment for severe acute malnutrition (SAM): a cluster-randomised trial in the Democratic Republic of the Congo

**DOI:** 10.1186/s12916-017-0848-y

**Published:** 2017-04-26

**Authors:** Emmanuel Grellety, Pélagie Babakazo, Amina Bangana, Gustave Mwamba, Ines Lezama, Noël Marie Zagre, Eric-Alain Ategbo

**Affiliations:** 1 Independent Consultant, Paris, France; 2 Kinshasa School of Public Health, University of Kinshasa, Kinshasa, Democratic Republic of the Congo; 3 United Nations International Children Emergency Fund, Kinshasa, Democratic Republic of the Congo; 4 Save the Children United Kingdom, Kinshasa, Democratic Republic of the Congo; 5 United Nations International Children Emergency Fund West and Central Africa Regional Office, Dakar, Senegal

**Keywords:** Malnutrition, Severe acute malnutrition, Cash transfer, Community-based management of acute malnutrition, CMAM, Democratic Republic of the Congo, Cluster-randomised trial

## Abstract

**Background:**

Cash transfer programs (CTPs) aim to strengthen financial security for vulnerable households. This potentially enables improvements in diet, hygiene, health service access and investment in food production or income generation. The effect of CTPs on the outcome of children already severely malnourished is not well delineated. The objective of this study was to test whether CTPs will improve the outcome of children treated for severe acute malnutrition (SAM) in the Democratic Republic of the Congo over 6 months.

**Methods:**

We conducted a cluster-randomised controlled trial in children with uncomplicated SAM who received treatment according to the national protocol and counselling with or without a cash supplement of US$40 monthly for 6 months. Analyses were by intention to treat.

**Results:**

The hazard ratio of reaching full recovery from SAM was 35% higher in the intervention group than the control group (adjusted hazard ratio, 1.35, 95% confidence interval (CI) = 1.10 to 1.69, *P* = 0.007). The adjusted hazard ratios in the intervention group for relapse to moderate acute malnutrition (MAM) and SAM were 0.21 (95% CI = 0.11 to 0.41, *P* = 0.001) and 0.30 (95% CI = 0.16 to 0.58, *P* = 0.001) respectively. Non-response and defaulting were lower when the households received cash. All the nutritional outcomes in the intervention group were significantly better than those in the control group. After 6 months, 80% of cash-intervened children had re-gained their mid-upper arm circumference measurements and weight-for-height/length Z-scores and showed evidence of catch-up. Less than 40% of the control group had a fully successful outcome, with many deteriorating after discharge. There was a significant increase in diet diversity and food consumption scores for both groups from baseline; the increase was significantly greater in the intervention group than the control group.

**Conclusions:**

CTPs can increase recovery from SAM and decrease default, non-response and relapse rates during and following treatment. Household developmental support is critical in food insecure areas to maximise the efficiency of SAM treatment programs.

**Trial registration:**

ClinicalTrials.gov, NCT02460848. Registered on 27 May 2015.

**Electronic supplementary material:**

The online version of this article (doi:10.1186/s12916-017-0848-y) contains supplementary material, which is available to authorized users.

## Background

Childhood malnutrition is a significant cause of ill health and poor development worldwide. High-quality nutrition is essential in early childhood to ensure healthy growth, proper organ formation and function, a strong immune system and neurological and cognitive development. Children with severe acute malnutrition (SAM) are at high risk of morbidity and death [1]. There are estimated to be 19 million children younger than 5 years of age with SAM worldwide, of whom more than 800,000 die annually [1].

Although considerable progress has been made in treating SAM [2–4], one way to reduce the burden of acute malnutrition is to prevent its emergence among children by increasing the resilience of poor and vulnerable households. Poverty is generally acknowledged to be the major antecedent of malnutrition [5–7], and social protection and safety-net interventions are important to protect maternal and child nutrition [8]. Recently, cash transfer programs (CTPs), which deliver direct unconditional or conditional cash to households, are being tested in developing countries [9]. Such programs have been used in developed countries for many years as the main method for poverty reduction and social security. Although literature reviews of CTPs used in humanitarian relief interventions have appeared over the last few years, none has provided conclusive evidence of a sustained positive impact on child nutritional status, and several comment that the ways in which these interventions have an impact are not clearly understood [10–17]. Even if an increasing number of studies have highlighted a positive effect of CTPs in increasing diet diversity [18–20], food consumption [20, 21], health status [18, 22] and access to health care [19, 21], the evidence of a resulting effect on child nutrition is mixed and inconclusive, particularly in sub-Saharan Africa [18, 19, 23]. Nevertheless, evidence is emerging to show that complementary cash interventions could improve the results of nutrition interventions. In Niger and Somalia, cash transfers provided to households with acutely malnourished children improved recovery beyond the provision of ready-to-use therapeutic foods (RUTF) [24, 25]. CTPs appear to reduce the sharing of therapeutic foods and may improve the effectiveness of such interventions. It is also possible that the additional cash could increase the cost-effectiveness of the nutrition intervention, if gains in effectiveness more than balance the added cost of the subvention.

There is a particular need to determine the effect of CTP strategies and their impact on vulnerable households with malnourished members among different target groups and contexts. Most underlying causes of malnutrition are a function of people’s resources and social context. What households produce as well as the time they have to care for dependent members are determined by a range of social, economic and political factors; these are thought to include the division of labour, gender inequality, educational opportunities and property and power relations. When households have more money, they can diversify their diets by buying or growing food of a higher quality, being able to afford to attend the health centre and investing capital to create ongoing income-generating opportunities. Here, we hypothesised that additional cash will have a direct effect to improve the final outcome of children enrolled in a community-based management of acute malnutrition (CMAM) program. Specifically, within the household the cash would decrease intra-household sharing of the RUTF and improve the food diversity and consumption. The cash would improve the outcome of the malnourished child by reducing death, morbidity, transfer to hospital, defaulting, relapse or other causes of failure. The child would have higher rates of mid-upper arm circumference (MUAC) and weight gain and derived anthropometric indices.

This paper presents the findings from a cluster-randomised trial comparing the outcome of a standard Outpatient Therapeutic Program (OTP) for SAM and infant and young child feeding (IYCF) counselling with and without a monthly cash transfer over a 6-month period in the Democratic Republic of the Congo (DRC).

## Methods

### Study design

The present study took place in the DRC, where around two million children younger than 5 years old are affected by SAM every year [26, 27]. This case load is higher than the number of children suffering from SAM in all of the Sahelian countries combined. Our cluster-randomised controlled trial was carried out in Bipemba (a commune of 57.5 km^2^ in the city of Mbuji-Mayi; GPS 6°11'S 23°54'E) in the Kasaï-Oriental province. There are 52 functional health centres where management of SAM was established using the OTP module of the National Integrated Management of Acute Malnutrition protocol. This setting was selected for the following reasons. First, the socioeconomic homogeneity of the whole livelihood zone was confirmed by three baseline surveys: (1) a market chain analysis [28], (2) a Household Economy Approach (HEA) assessment [29] and (3) a Knowledge, Attitude and Practices (KAP) survey [30]. Second, the prevalence of severe wasting, using World Health Organisation (WHO) criteria, in children between 6 to 59 months of age reported in the province between 2013 and 2014 was above 2% [26]. Third, there are no major seasonal changes in this area which could affect the outcome of children discharged at different times during the study [31].

A cluster was defined by a health centre and its catchment area. There were 52 eligible health centres, of which 20 were selected. The health centres were selected at random by sequentially drawing, in public, sealed, numbered papers from a basket in the presence of all 52 health centre representatives. A priori, in order to minimise contamination bias between clusters if a subsequent health centre was drawn but was found to have a catchment area adjoining a health centre that had already been selected, that centre was eliminated from the draw and a further sealed paper drawn; the health centre representatives were aware of this procedure before the draw took place. When the 20 health centres that were to be included in the trial were chosen, a second selection round was conducted with each selected centre drawing a sealed paper with either a 1 or 2 written on it to indicate to which arm of the trial that centre would be entered. A third selection was made in private when one of the arms was assigned at random to receive standard SAM management plus counselling and the other arm standard SAM management plus counselling plus cash transfer. In order to detect an expected recovery rate of 70% after 8 weeks and an expected difference between groups of 10%, with an α-error = 5%, a β-error = 20% and an intra-class correlation of 0.001, a sample size of 1392 children was required. Assuming a study dropout of up to 15%, a total sample size of 1600 children was projected with an average of 80 participants per cluster: 800 per arm. Because we wished to avoid any confounding from temporal variation in the centres’ recruitment rate, we determined the potential case load from the admissions during the previous year when there had not been any active screening of the population. We then conducted an exhaustive, active screening of the whole catchment area of each health centre in order to recruit the required number of children (about 4 per centre per day) over as short a time as possible. The staff in each health centre comprised the nurse in charge and two Save the Children staff dedicated to check and collect the data. The study’s staff were supervised by Pronanut (the DRC Government nutrition agency) and UNICEF. The study teams were trained intensively for 3 weeks prior to the start of the study by the Principal Investigator, and the details of the national protocol were revised with the nurses in charge of the centres.

Inclusion criteria for the trial required participants to be eligible for outpatient SAM treatment according to the integrated management of acute malnutrition (IMAM) national protocol [32], have no congenital abnormality that may affect growth, no peanut allergy, be resident in the catchment area of the health centre and be available for the 6-month study period. A child was eligible for selection if aged between 6 and 59 months with a MUAC <115 mm and/or a weight-for-height/length Z-score (WHZ) < –3Z [33] and/or with bilateral oedema. As treatment was as an outpatient at recruitment, the child also had to have a moderate or good appetite using a formal test meal of RUTF, an absence of defined clinical complications and no generalised (grade +++) oedema according to the national protocol [32]. Patients with complications, a poor appetite or generalised oedema were referred for in-patient management and were not subsequently enrolled after discharge. Patients who deteriorated after recruitment were maintained within the study; if they required admission to hospital, they were again followed after discharge. The participant children were either detected through passive screening during routine growth monitoring and outpatient clinic visits or through active screening in the community using MUAC and oedema only; they were then sequentially included in the trial until the preset sample size was reached.

### Ethical considerations

The objectives and procedures of the study were explained to heads of households or principal child caregivers before inclusion. An informed consent statement was read aloud in the local dialect before consenting adults signed or gave their fingerprint. It was emphasised that participation in the study was not a pre-condition for obtaining nutritional treatment and free medical services. It was clearly stated that participants were free to withdraw from the study at any time. The protocol was approved by both the National Ethical Committee of the School of Public Health from the Faculty of Medicine of University of Kinshasa and the Ministry of Public Health. The study was registered on ClinicalTrials.gov as NCT02460848 and was performed in accordance with Good Clinical Practice (GCP) guidelines for clinical trials and according to the tenets of the Declaration of Helsinki.

### Interventions

In both study arms, children with SAM received treatment according to the IMAM national protocol for OTP and counselling on IYCF [32]. At admission, children received a weekly take-home ration of RUTF supplying approximately 170 kcal per kilogram per day (Plumpy’nut®, Nutriset) and routine medicines: routine anti-helminthic treatment for those older than 1 year of age, 7 days of amoxicillin and a vitamin A preventive dose; where indicated measles vaccination and anti-malarial treatment were given. Weekly follow-up was conducted at the health centres. At baseline and at each visit the following were performed: clinical history; physical examination; an appetite test; and anthropometric measurements using standard techniques (weight was measured with UNISCALE electronic scales with a precision of 100 g; length with a rigid wooden length board for children <87 cm and standing height for children ≥87 cm to the nearest 0.1 cm; MUAC was measured with a non-stretchable tape with a precision of 1 mm) [34]. They were then given the next therapeutic ration until they reached discharge criteria. Children were transferred to inpatient therapeutic program (ITP) for any clinical complication, including poor appetite, or meeting the ‛failure to respond to treatment’ criteria of the national protocol. Children were discharged as ‛recovered’ if they achieved WHZ ≥ −1.5 (WHO Growth Standards 2006) or MUAC ≥125 mm at two consecutive visits and absence of bilateral oedema for at least 14 days. Non-response was defined as not meeting the criteria for nutritional recovery by 12 weeks and default as failing to appear for two consecutive follow-up visits confirmed by a home visit. Diet replacement and intra-household sharing of the RUTF were assessed using a questionnaire with the household each week during the treatment period. The IYCF counselling sessions took place in the household during a home visit; they lasted approximately 1.5 h at admission and 45 min during subsequent visits. During the first 3 months, caretakers were also invited to attend cooking sessions where recipes for optimising the children’s meals with local ingredients were demonstrated.

After discharge, children were followed up monthly: medical history, physical examination, and anthropometry were repeated at each visit until the end of the study. Observations were concluded 6 months after recruitment. Thus, those who were under treatment for longer had a shorter post-discharge follow-up; as failure to respond was defined as still being malnourished after 12 weeks of treatment, the minimum follow-up period was 14 weeks, so that individual children had either three, four or five monthly follow-up assessments. Children were defined as having relapsed to SAM if they again reached any of the three criteria defining SAM at least once during the monthly follow-up visits after being discharged as recovered. Children’s relapse to moderate acute malnutrition (MAM) was defined as the development of WHZ < –2.0 and ≥ –3.0 (WHO Growth Standards 2006) or MUAC <125 mm and ≥115 mm (without bilateral oedema) at least once during the monthly follow-up visits, without the child developing SAM criteria during any other follow-up visit. ‘Unknown’ was defined as a defaulter not confirmed by a home visit or with no information for the child at the end of the trial. Withdrawal from the study was defined as participants who elected to stop the study for personal reasons.

At enrolment, trained health workers recorded the socioeconomic characteristics of the household and categorised it into wealth groups according to local definitions of wealth and assets from the HEA assessment. The Household Dietary Diversity Scores (HDDSs) [35] and the Food Consumption Scores (FCSs) [36] were collected at the first and last household visit; each of them included respectively 12 and 8 food groups. Individual Dietary Diversity Scores (IDDSs) [35, 37] included 8 food groups and were collected at each clinic visit, i.e. weekly during treatment and monthly during follow-up.

All participating caretakers from the intervention group with one or more children with SAM received an unconditional cash transfer of US$40 value each month during treatment and follow-up for a total of 6 months (US$240 in total). We emphasised to the participants that they could use the funds in any way they saw fit in a completely unrestricted way without any conditions being imposed on how the cash was used. The cash was distributed directly to the child’s caretaker for each household in the intervention group without informing or involving the health centre staff. Each month the cash was given from 10 separate administrative offices (separately located from the health centre) by two Save the Children financial staff and two food security supervisors attached to the study and completely independent of the health centre staff. The dispensing of the money was spread throughout the month according to the patient's admission date, so that daily attendance was minimised. This mechanism was considered the most secure to preserve confidentiality and to avoid contamination bias; electronic and other forms of cash transfer were not available in the area at the time of this study.

The amount given to each household was fixed and not adjusted by the size of the household or the number of malnourished children. This amount was calculated to provide 70% of the monthly household income for a household of seven persons characterised as very poor using HEA criteria [29]. Seventy percent of the average monthly expenditure would be sufficient to purchase all the household’s food energy needs (2100 kcal per day per person), the costs of food preparation and consumption (e.g. salt, soap, kerosene and/or firewood and basic lighting) and drinking water. The main household expenditures and sources of income were collected at enrolment; how the household spent the additional cash provided by the CTP was determined monthly during the study.

The cash transfer for a family of seven persons amounts to 18 cents US per person per day. As the area is isolated without reliable ground transportation of goods, it tends to be more expensive than less isolated areas. The cash given in other CTPs in the DRC varies from US$110 to US$135 monthly (except for one pilot project which dispensed US$20.5 per month). The amount given in this study was judged by the Emergency Department of UNICEF to be a sustainable amount that could be supported by donors and other stakeholders if the program were to be scaled up; the higher amounts given in other programs were judged to be unsustainable operationally [38].

### Data analysis

All data were collected on standardised paper forms and double-entered into EpiData version 3.1 (EpiData Association) by staff unaware of the arm to which each health centre belonged. Any anthropometric data which fell outside the limits of biological plausibility, using WHO criteria, were eliminated from the database [39]. Changes in height of more than 5 cm, weight by more than 10% of body weight or MUAC of more than 20 mm between visits were considered implausible data recording or measurement errors; in this case, the data from the previous or the later visit were substituted. All data cleaning was performed by a qualified individual who was not aware of the assignment, grouping or centres to which individual children belonged. Potential anthropometric errors which were slightly less gross were considered ‛suspect’ and viewed individually in relation to the other longitudinal values of that variable obtained with the rest of the child’s data blinded in accordance with recent recommendations [40]. Obvious errors inconsistent with the other recordings of that variable were corrected (such as a single value showing a loss of height with a subsequent measurement in agreement with the antecedent measurements), but other forms of suspect data were not altered. All analyses were by intention to treat at recruitment except the examination of relapse, which was confined to children who reached the discharge criteria as ‛recovered’ (children who had not recovered could not relapse). Statistical significance was set at the 5% level. All analyses were conducted in accordance with Consolidated Standards of Reporting Trials (CONSORT) guidelines, extended to cluster-randomised trials, using R software version 3.2.2.

Significance testing for differences between intervention and control groups at baseline was performed using the independent sample Student's *t* test for continuous variables and the chi-square test for categorical variables.

Differences in child recovery between trial arms of the primary outcomes and relapse rate after discharge from therapeutic home treatment were tested by using a mixed-effects Poisson regression model, with health centre as random effect to estimate the incidence rate ratio (IRR). Next, we estimated hazard ratios (HRs) and 95% confidence intervals (CIs) using marginal Cox proportional hazards models adjusted for baseline values, where the outcome variable is time from recruitment to the event (recovery) and the time scale is calendar week. All 95% CIs used robust estimates of the variance to account for clustering at the health centre level as well as a shared-frailty model as developed by Andersen [41]. Potential interactions were assessed using a partial likelihood ratio test and a robust score test to calculate *P* values. We checked for possible deviation from the proportional hazards assumption of the Cox regression model by using the non-proportionality test on the basis of the Schoenfeld residuals.

Comparisons between arms for the secondary outcomes (recovery, defaulter, transfer, death and relapse rates), time to recovery, length/height change, IDDS, HDDS, FCS and daily, weekly and monthly anthropometrics changes were made by using linear mixed-effects models for continuous outcome variables, whereas mixed-effects logistic regression models were used for proportions, with health centre as random effect and models adjusted for baseline values. Analyses of anthropometric data which depended upon body weight excluded children with oedema; oedematous children were included in all other analyses.

We produced and used Kaplan-Meier plots to estimate the probability of failure to achieve and maintain nutritional recovery up to 6 months from enrolment. Survival curves of two groups were compared using the Cox regression analyses with robust estimates of the variance to account for clustering at the health centre level, and *P* values were computed with the robust score test.

## Results

**Fig. 1:**
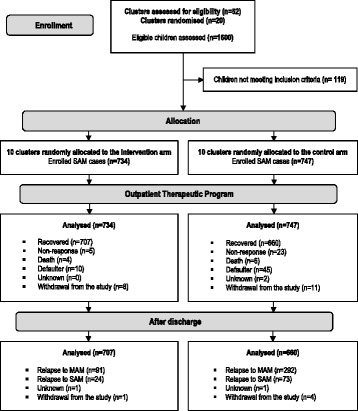
Trial flow chart of the study. *Recovery* was defined as a WHZ ≥ −1.5 (WHO Growth Standards 2006) or MUAC ≥125 mm at two consecutive visits and absence of bilateral oedema for at least 14 days. *Non-response* was defined as not meeting the criteria for nutritional recovery at 12 weeks and default as failing to appear for two consecutive follow-up visits confirmed by a home visit. *Defaulter* was defined as a patient absent for two consecutive visits and confirmed as absent by a home visit at week 3. *Relapse to MAM* was defined as the development of a WHZ < –2.0 and ≥ –3.0 (WHO Growth Standards 2006) or MUAC <125 mm and ≥115 mm (without bilateral oedema) at least once during the monthly follow-up visits without the child developing SAM criteria during any other follow-up visit. *Relapse to SAM* was defined as development of a WHZ < −3.0 (WHO Growth Standards 2006) or MUAC <115 mm or presence of bilateral oedema at least once during the monthly follow-up visits. *Unknown* was defined as defaulter not confirmed by a home visit or as no information for children at the end of the trial. *Withdrawal* from the study was defined as participants who had to stop the study for personal reasons

Between 16 July 2015 and 31 July 2015, 1600 children were admitted to the centres with a diagnosis of SAM; 119 (7.4%) children did not meet the inclusion criteria and were excluded. Among those, some were admitted using the IMAM unisex weight-for-height table but were ineligible using sex-specific assessment, a few lived outside the catchment area and others were referred directly to the hospital. Figure 1 shows the flow and outcomes of the 1481 subjects in the two arms of the study.

The control and intervention (cash) group’s baseline characteristics are shown and compared in Table 1. The malnourished children in each group were not significantly different; in particular, the anthropometry was similar between the groups. The IDDS becomes non-significant if a correction for multiple significance tests is made *P* > 0.05 (but the IDDS was used for the adjusted Cox analyses). The mothers had a higher school achievement in the intervention group, but were otherwise not different. The HEA assessment showed that 75% of the households were classified as poor or very poor in both arms. The household size and number of children younger than 5 years were both greater in the intervention group. However, there were fewer household members younger than 5 years in the intervention group compared to the control group (5.3 vs 5.5), so that the increased number of young children, and hence the dependency ratio, in the intervention group would tend to make this group more vulnerable to nutritional deficits. In both groups, less than 20% of the households had an acceptable diet diversity score with 43% being in the lowest category; 53% were considered to have poor or borderline food consumption. These results are consistent with previous assessments conducted in Kasaï-Oriental [42] and show that the households with SAM children are generally poor with a restricted diet, and most had an unacceptable food consumption score. The baseline characteristics of all enrolled children appeared balanced between the trial arms.

**Table 1:** Child, maternal and household characteristics at inclusion by study group

Characteristics	Intervention arm		Control arm		*P* value^a^
Number (%)	734	(49.6)	747	(50.4)	
Child characteristics					
Sex, female, *n* (%)	422	(57.5)	429	(57.4)	1.000
Age (months), mean (SD)	27.7	±14.7	27.0	±13.8	0.348
Age category, *n* (%)					0.973
6–11 months	94	(12.8)	94	(12.6)	
12–23 months	213	(29.0)	214	(28.6)	
24–59 months	427	(58.2)	439	(58.8)	
Sibling rank, *n* (%)					0.144
Elder	86	(11.7)	69	(9.2)	
Second	103	(14.0)	109	(14.6)	
Third	126	(17.2)	119	(15.9)	
Fourth	130	(17.7)	114	(15.3)	
Fifth and more	288	(39.2)	336	(45.0)	
Do not know	1	(0.1)	0	(0.0)	
Weight (kg), mean (SD)^b^	7.6	±1.7	7.5	±1.7	0.442
Height/length (cm), mean (SD)	76.8	±9.3	76.3	±9.1	0.311
WHZ, mean (SD)^b^	–3.1	±0.6	–3.0	±0.7	0.052
HAZ, mean (SD)	–3.4	±1.4	–3.5	±1.5	0.342
MUAC (mm), mean (SD)^b^	112.3	±4.6	111.9	±5.6	0.086
Bilateral oedema, n (%)	63	(8.6)	88	(11.8)	0.052
IDDS, mean (SD)	2.8	±1.0	2.7	±0.9	0.009
Maternal characteristics					
Age (years), mean (SD)	29.5	±7.7	29.6	±7.4	0.675
School achievement, *n* (%)					<0.001
None	53	(7.2)	47	(6.3)	
Primary school	369	(50.3)	466	(62.4)	
Secondary or high	286	(39.0)	211	(28.2)	
Do not know	26	(3.5)	23	(3.1)	
Marital status, *n* (%)					0.649
Monogamous marriage	505	(68.8)	513	(68.7)	
Polygamous marriage	166	(22.6)	173	(23.2)	
Other (single, widow, divorcee, separated)	40	(5.4)	45	(6.0)	
Do not know	23	(3.1)	16	(2.1)	
BMI, mean (SD)	21.1	±2.8	21.0	±2.7	0.423
Breastfeeding at time of enrolment, *n* (%)	243	(33.1)	240	(32.1)	0.729
Household characteristics					
Household size, mean (SD)	8.0	±2.5	7.6	±2.5	0.001
No. of children <5 years at home, mean (SD)	2.7	±1.3	2.1	±0.9	<0.001
Socioeconomic level (HEA criteria), *n* (%)					0.058
Very poor	378	(51.5)	371	(49.7)	
Poor	184	(25.1)	179	(24.0)	
Middle	68	(9.3)	103	(13.8)	
Better-off	104	(14.2)	94	(12.6)	
HDDS, mean (SD)	4.1	±1.5	4.0	±1.5	0.185
HDDS category, *n* (%)					0.098
Lowest dietary diversity (≤3 food groups)	296	(40.3)	339	(45.4)	
Medium dietary diversity (4–5 food groups)	309	(42.1)	299	(40.0)	
High dietary diversity (≥6 food groups)	129	(17.6)	109	(14.6)	
FCS, mean (SD)	43.7	±12.19	43.5	±12.8	0.768
FCS category, *n* (%)					0.676
Poor (score 0–28)	59	(8.0)	68	(9.1)	
Borderline (score 28.5–42)	311	(42.4)	322	(43.1)	
Acceptable (score >42)	364	(49.6)	357	(47.8)	

^a^From Student’s *t* tests for continuous variables and chi-square tests for categorical variables. Note: no corrections for multiple significance tests have been made in this table. With Bonferroni-Holm correction the child’s IDDS score becomes non-significant. It was however, retained as an adjustment in the Cox analyses; the other significances were not changed

^b^Children with oedema were excluded from the analysis for the assessment of weight and WHZ

*HAZ* height-for-age Z-score, *BMI* body mass index

The results of the treatment are given in Table 2. The difference in the proportion of children reaching the recovery criteria was highly significant between the control and intervention groups. This was partly due to children who failed to respond to treatment and to defaulting being significantly lower for children whose households received the cash supplement than those who did not. There was no difference in the mortality rate; however, the number of deaths recorded was very low and insufficient to show a statistical difference.

**Table 2:** Outcomes and progress of children with SAM during treatment by study group

Parameters	Intervention arm		Control arm		*P* value^a^
Number (%)	734	(49.6)	747	(50.4)	
Recovery, *n* (%)	707	(96.3)	660	(88.4)	<0.001
Non-response, *n* (%)	5	(0.7)	23	(3.1)	0.016
Defaulter, *n* (%)	10	(1.4)	45	(6.0)	<0.001
Death, *n* (%)	4	(0.5)	6	(0.8)	0.759
Length of stay (week), mean (SD)	6.77	±2.31	7.04	±2.77	0.541
Length/height change (mm/week), mean (SD)	0.55	±1.15	0.39	±1.18	0.554
HAZ change (Z-score/month), mean (SD)	–0.15	±0.24	–0.18	±0.23	0.278
Weight change (gram/kg/day), mean (SD)^b^	4.37	±1.88	3.84	±1.98	0.021
WAZ change (Z-score/month), mean (SD)^b^	0.79	±0.39	0.67	±0.41	0.011
WHZ change (Z-score/month), mean (SD)^b^	1.41	±0.57	1.25	±0.64	0.026
BMIZ change (Z-score/month), mean (SD)^b^	1.49	±0.60	1.33	±0.68	0.038
MUAC change (mm/day), mean (SD)	0.30	±0.13	0.29	±0.16	0.552
MUACZ-age change (Z-score/month), mean (SD)	0.91	±0.39	0.86	±0.49	0.493
MUACZ-ht change (Z-score/month), mean (SD)	0.95	±0.41	0.92	±0.51	0.604
IDDS change, mean (SD)	1.30	±1.54	0.42	±1.29	0.008

^a^Comparisons between arms were made by using linear mixed-effects models for continuous outcomes, whereas mixed-effects logistic regression models were used for proportions, with health centre as random effects

^b^Children with oedema were excluded from the analysis of parameters including weight

*HAZ* height-for-age Z-score, *WAZ* weight-for-age Z-score, *BMIZ* body mass index for age Z-score, *MUACZ-age* mid-upper arm circumference for age Z-score, *MUACZ-ht* mid-upper arm circumference for height Z-score

During treatment, changes in weight, WHZ, weight-for-age Z-score (WAZ), body mass index Z-score (BMIZ) and IDDS were all significantly greater in the intervention group compared to the control group; however, there was not a difference in the rate of increase in MUAC or height. Both groups gained less height than expected when compared with the standards for age of height-for-age Z-score (HAZ); the changes were not significant. The mean length of stay among children in the therapeutic program was 6.9 weeks (±2.5). It did not differ significantly between the groups, although the median was 1 week shorter in the intervention group.

The Cox regression analyses (Table 3) showed that by week 8 of treatment the cumulative probability of recovery was 29% higher in the intervention group than the control group; it was 28% at week 10 and increased to 35% by the 12^th^ week. Adjustment of the analysis for the significant baseline household and maternal characteristics increased the difference between the groups by a trivial amount. The mixed-effects Poisson regression analysis gave similar results.

**Table 3:** Cumulative recovery rate during therapeutic home treatment for SAM by study group

Nutritional recovery^a^	Intervention arm	Control arm	*P* value
Week 6			
Number^b^	734	747	
Number of events/child-weeks at risk	364/4000	307/3979	
Incidence rate per 100 child-weeks (95% CI)	9.10 (8.17–10.00)	7.71 (6.85–8.58)	
Incidence rate ratio (95% CI)^c^	1.18 (0.78–1.80)	1.00 (Reference)	0.414
Unadjusted HR (95% CI)	1.21 (0.80–1.81)	1.00 (Reference)	0.372
Adjusted HR (95% CI)^d^	1.20 (0.78–1.81)	1.00 (Reference)	0.184
Week 8			
Number^b^	734	747	
Number of events/child-weeks at risk	539/4592	455/4681	
Incidence rate per 100 child-weeks (95% CI)	11.74 (10.75–12.73)	9.72 (8.83–10.61)	
Incidence rate ratio (95% CI)^c^	1.18 (0.88–1.58)	1.00 (Reference)	0.128
Unadjusted HR (95% CI)	1.29 (0.96–1.74)	1.00 (Reference)	0.084
Adjusted HR (95% CI)^d^	1.29 (1.01–1.68)	1.00 (Reference)	0.027
Week 10			
Number^b^	734	747	
Number of events/child-weeks at risk	645/4874	587/5081	
Incidence rate per 100 child-weeks (95% CI)	13.23 (12.21–14.25)	11.55 (10.62–12.49)	
Incidence rate ratio (95% CI)^c^	1.14 (1.01–1.27)	1.00 (Reference)	0.039
Unadjusted HR (95% CI)	1.27 (1.01–1.60)	1.00 (Reference)	0.046
Adjusted HR (95% CI)^d^	1.28 (1.01–1.62)	1.00 (Reference)	0.011
Week 12			
Number^b^	734	747	
Number of events/child-weeks at risk	707/4970	660/5260	
Incidence rate per 100 child-weeks (95% CI)	14.23 (13.18–15.27)	12.54 (11.59–13.50)	
Incidence rate ratio (95% CI)^c^	1.15 (1.03–1.27)	1.00 (Reference)	0.020
Unadjusted HR (95% CI)	1.34 (1.09–1.66)	1.00 (Reference)	0.026
Adjusted HR (95% CI)^d^	1.35 (1.10–1.69)	1.00 (Reference)	0.007

^a^Recovery was defined as a WHZ ≥ −1.5 (WHO Growth Standards 2006) or MUAC ≥125 mm at two consecutive visits and absence of bilateral oedema for at least 14 days

^b^Number of children contributing to unadjusted analysis

^c^Computed by using a mixed-effects Poisson regression model, with health centre as random effects

^d^From marginal Cox proportional hazards models where the outcome variable is time until first event and time is calendar week. 95% CIs used robust estimates of the variance to account for clustering at the health centre level, and *P* value was performed with the robust score test. Co-variates in the adjusted model included the household size, the IDDS score and the school achievement of the mother

Table 4 shows the follow-up data of the children after recovery. At this stage the control children were not receiving any further input, but the intervention group continued to receive US$ 40/month. Compared to the control group, the cash-intervention group shows that the children from households with additional funds continued to have a higher weight and MUAC gains, which resulted in significant continued catch-up. The WAZ, WHZ, BMIZ, mid-upper arm circumference for age Z-score (MUACZ-age) and mid-upper arm circumference for height Z-score (MUACZ-ht) changes are all significantly greater than zero, showing true catch-up compared to the WHO standards. In contrast, the control group’s changes in Z-score for these parameters were all negative; this was significantly less than no change for WHZ, and not different from zero for the other indices. This shows that, on average, the control children were deteriorating after discharge to become progressively less well-nourished with time after recovery and discharge from the treatment program. There was no catch-up in height-for-age for either group, so that the children remained stunted and the intervention did not improve linear growth over the time course of this study. When MUAC is related to height, there was also considerable catch-up in the intervened children compared with the control children.

**Table 4:** Results during follow-up after discharge among those who recovered by study group

Parameters	Intervention arm		Control arm		*P* value^a^
Number (%)	707	(51.7)	660	(48.3)	
Relapse to MAM, *n* (%)^b^	91	(12.9)	292	(44.2)	<0.001
Relapse to WHZ < –2 and ≥ –3	58	(8.2)	201	(30.5)	
Relapse to MUAC <125 mm and ≥115 mm	57	(8.1)	279	(42.3)	
Relapse to SAM, *n* (%)^c^	24	(3.4)	73	(11.1)	<0.001
Relapse to WHZ < -3	8	(1.1)	30	(4.5)	
Relapse to MUAC <115 mm	0	(0.0)	22	(3.3)	
Relapse to oedema	16	(2.3)	26	(3.9)	
Length/height change (mm/week), mean (SD)	1.35	±1.33	1.45	±0.18	0.602
Weight change (gram/kg/day), mean (SD)^d^	0.86	±0.66	0.46	±0.72	<0.001
MUAC change (mm/day), mean (SD)	0.060	±0.05	0.012	±0.04	<0.001
HAZ change (Z-score/month), mean (SD)	–0.018**	±0.18	0.003	±0.15	0.502
WAZ change (Z-score/month), mean (SD)^d^	0.08**	±0.15	–0.01	±0.15	<0.001
WHZ change (Z-score/month), mean (SD)^d^	0.13**	±0.24	–0.03**	±0.22	0.006
BMIZ change (Z-score/month), mean (SD)^d^	0.14**	±0.28	– 0.02*	±0.23	0.017
MUACZ-age change (Z-score/month), mean (SD)	0.132**	±0.14	– 0.001	±0.14	<0.001
MUACZ-ht change (Z-score/month), mean (SD)	0.141**	±0.14	0.005	±0.15	<0.001

*P < 0.05 for change in standardised anthropometric parameter different from zero

**P < 0.001 for change in standardised anthropometric parameter significantly different from zero

^a^Comparisons between arms were made by using linear mixed-effects models for continuous outcomes, whereas mixed-effects logistic regression models were used for proportions, with health centre as random effects

^b^Relapse to MAM was defined as the development of a WHZ < –2.0 and ≥ –3.0 (WHO Growth Standards 2006) or MUAC <125 mm and ≥115 mm (without bilateral oedema) at least once during the monthly follow-up visits without the child developing SAM criteria during any other follow-up visit

^c^Relapse to SAM was defined as development of a WHZ < −3.0 (WHO Growth Standards 2006) or MUAC <115 mm or presence of bilateral oedema at least once during the monthly follow-up visits

^d^Children with oedema were excluded from the analysis of parameters including weight

This deterioration in the control children’s status post-discharge is confirmed by the proportion of children who again developed MAM or SAM. Altogether 44% of the control children deteriorated after treatment, 11% to a level where they would be readmitted for SAM. Most of the children who relapsed to MAM did so with both WHZ and MUAC. Furthermore, as there was no significant gain in height, the deterioration in WHZ for those who relapsed to MAM or to SAM could not be ascribed to a disproportionate gain in height relative to weight.

For those who relapsed to SAM, only 8 of the 707 (1.1%) intervened children relapsed anthropometrically by the WHZ criterion and none by the MUAC criterion. The main type of relapse in the intervened children was re-development of oedema. There were slightly more children who relapsed with oedema in the control group (26 vs 16), but the difference was not significant (*χ* ^2^ = 2.7, *P* = 0.10). In contrast, 47 (7.1%) of the control children re-developed anthropometric SAM having been discharged as cured.

The multivariate analysis results of the relative risk of relapse are shown in Table 5. Here the data are adjusted for baseline variables to take into account the initial food consumption score, the household size and the education of the mother to determine whether differences in the households could account for the difference. The cluster effect was also allowed for in case the performance of the different health centres could account for the differences. The results remained highly significant for relapse to MAM, with an IRR of 0.25 for the intervention group and a HR of 0.21. The more serious relapse to SAM was also highly significant, being less than one third for both IRR and HR, despite our including the relapse to oedema in the model. For this analysis it should be noted that the reference group for those who relapsed to SAM included all children who remained normal *and* those who relapsed to MAM on the basis that those who developed MAM were also at risk of further deterioration to SAM. If the children who developed oedema are omitted from the analysis and we include only those who relapsed anthropometrically, the incidence rate and hazard ratios are even lower. Adjustment for the baseline characteristics had almost no effect upon the results, confirming that the two groups were balanced in terms of child, maternal and household characteristics.

**Table 5:** Relapse rate after discharge from therapeutic home treatment for SAM by study group

Nutritional relapse^a^^,^^b^	Intervention arm	Control arm	*P* value
Number (%)	707 (51.7)	660 (48.3)	
Relapsed to MAM^a^			
Number of events/child-weeks at risk	91/11,505	292/7813	
Incidence rate per 100 child-weeks (95% CI)	0.79 (0.63–0.95)	3.74 (3.31–4.17)	
Incidence rate ratio (95% CI)^c^	0.25 (0.14–0.45)	1.00 (Reference)	<0.001
Unadjusted HR (95% CI)	0.21 (0.11–0.41)	1.00 (Reference)	0.005
Adjusted HR (95% CI)^d^	0.21 (0.11–0.41)	1.00 (Reference)	0.001
Relapsed to SAM^b^			
Number of events/child-weeks at risk	24/12,761	73/11,558	
Incidence rate per 100 child-weeks (95% CI)	0.19 (0.11–0.26)	0.63 (0.49–0.78)	
Incidence rate ratio (95% CI)^c^	0.31 (0.19–0.49)	1.00 (Reference)	<0.001
Unadjusted HR (95% CI)	0.31 (0.17–0.59)	1.00 (Reference)	0.003
Adjusted HR (95% CI)^d^	0.30 (0.16–0.58)	1.00 (Reference)	0.001

^a^Relapse to MAM was defined as the development of a WHZ < –2.0 and ≥ –3.0 (WHO Growth Standards 2006) or MUAC <125 mm and ≥115 mm (without bilateral oedema) at least once during the monthly follow-up visits without the child developing SAM criteria during any other follow-up visit

^b^Relapse to SAM was defined as development of a WHZ < −3.0 (WHO Growth Standards 2006) or MUAC <115 mm or presence of bilateral oedema at least once during the monthly follow-up visits

^c^Computed by using a mixed-effects Poisson regression model, with health centre as random effects

^d^From marginal Cox proportional hazards models where the outcome variable is time until first event and time is calendar week. 95% CIs used robust estimates of the variance to account for clustering at the health centre level, and *P* value was performed with the robust score test. Co-variates in the adjusted model included the household size, the IDDS score and the school achievement of the mother

In Table 6 we present the changes over the whole study period from admission of the severely malnourished child to the end of the study 6 months later, by intention to treat. With the exception of height gain (and HAZ), *all* the anthropometric indices showed a highly significant benefit for the cash-intervention group compared to the control group. The IRR and the Cox analysis are given in Table 7 and confirm that the HR for the intervened children was less than three quarters of that for the children whose families did not receive the cash supplement.

**Table 6:** Overall changes over the 6-month period by study group (intention to treat)

Parameters	Intervention arm		Control arm		*P* value^a^
Number (%)	734	(49.6)	747	(50.4)	
Length/height change (mm/week), mean (SD)	1.14	±1.00	1.17	±0.88	0.644
HAZ change (Z-score/month), mean (SD)	–0.05	±0.15	–0.04	±0.12	0.578
Weight change (gram/kg/day), mean (SD)^b^	1.86	±0.68	1.41	±0.74	<0.001
WAZ change (Z-score/month), mean (SD)^b^	0.25	±0.11	0.16	±0.12	<0.001
WHZ change (Z-score/month), mean (SD)^b^	0.45	±0.18	0.30	±0.18	0.001
BMIZ change (Z-score/month), mean (SD)^b^	0.48	±0.21	0.33	±0.20	0.005
MUAC change (mm/day), mean (SD)	0.12	±0.04	0.08	±0.04	<0.001
MUACZ-age change (Z-score/month), mean (SD)	0.33	±0.11	0.22	±0.12	<0.001
MUACZ-ht change (Z-score/month), mean (SD)	0.35	±0.11	0.24	±0.12	<0.001
IDDS change, mean (SD)	1.53	±1.44	0.59	±1.24	0.013
HDDS change, mean (SD)	2.34	±1.91	0.44	±1.41	<0.001
HDDS category, *n* (%)					<0.001
Lowest dietary diversity (≤3 food groups)	66	(9.0)	194	(26.0)	
Medium dietary diversity (4–5 food groups)	205	(27.9)	358	(47.9)	
High dietary diversity (≥6 food groups)	439	(59.8)	126	(16.9)	
Lost to follow-up^c^	24	(3.3)	69	(9.2)	
FCS change, mean (SD)	19.9	±13.1	5.7	±13.1	<0.001
FCS category, *n* (%)					<0.001
Poor (score 0 - 28)	2	(0.3)	11	(1.5)	
Borderline (score 28.5–42)	26	(3.5)	175	(23.4)	
Acceptable (score >42)	682	(92.9)	492	(65.9)	
Lost to follow-up^c^	24	(3.3)	69	(9.2)	

^a^Comparisons between arms were made by using linear mixed-effects models for continuous outcomes, whereas mixed-effects logistic regression models were used for proportions, with health centre as random effects

^b^Children with oedema were excluded from the analysis of parameters including weight

^c^Ninety-three participants were dead, defaulters, unknown or withdrew from the study

**Table 7:** Failure to achieve and maintain nutritional recovery over a 6-month period (intention to treat)

Failure of nutritional recovery^a^	Intervention arm	Control arm	*P* value
Number (%)^b^	734 (49.6)	747 (50.4)	
Number of events/child-weeks at risk	144/16,408	457/13,257	
Incidence rate per 100 child-weeks (95% CI)	0.88 (0.73–1.02)	3.45 (3.13 – 3.76)	
Incidence rate ratio (95% CI)^c^	0.31 (0.21–0.46)	1.00 (Reference)	<0.001
Unadjusted HR (95% CI)	0.24 (0.15–0.39)	1.00 (Reference)	<0.001
Adjusted HR (95% CI)^d^	0.24 (0.15–0.39)	1.00 (Reference)	<0.001

^a^Failure to achieve and maintain nutritional recovery during treatment and follow-up to 6 months from enrolment was defined as dead, non-response to treatment after 12 weeks, a defaulter, a relapse to either SAM or MAM or unknown at 6 months

^b^Number of children contributing to unadjusted analysis

^c^Computed by using a mixed-effects Poisson regression model, with health centre as random effects

^d^From marginal Cox proportional hazards models where the outcome variable is time until first event and time is calendar week. 95% CIs used robust estimates of the variance to account for clustering at the health centre level, and *P* value was performed with the robust score test. Co-variates in the adjusted model included the household size, the IDDS score and the school achievement of the mother

**Fig. 2:**
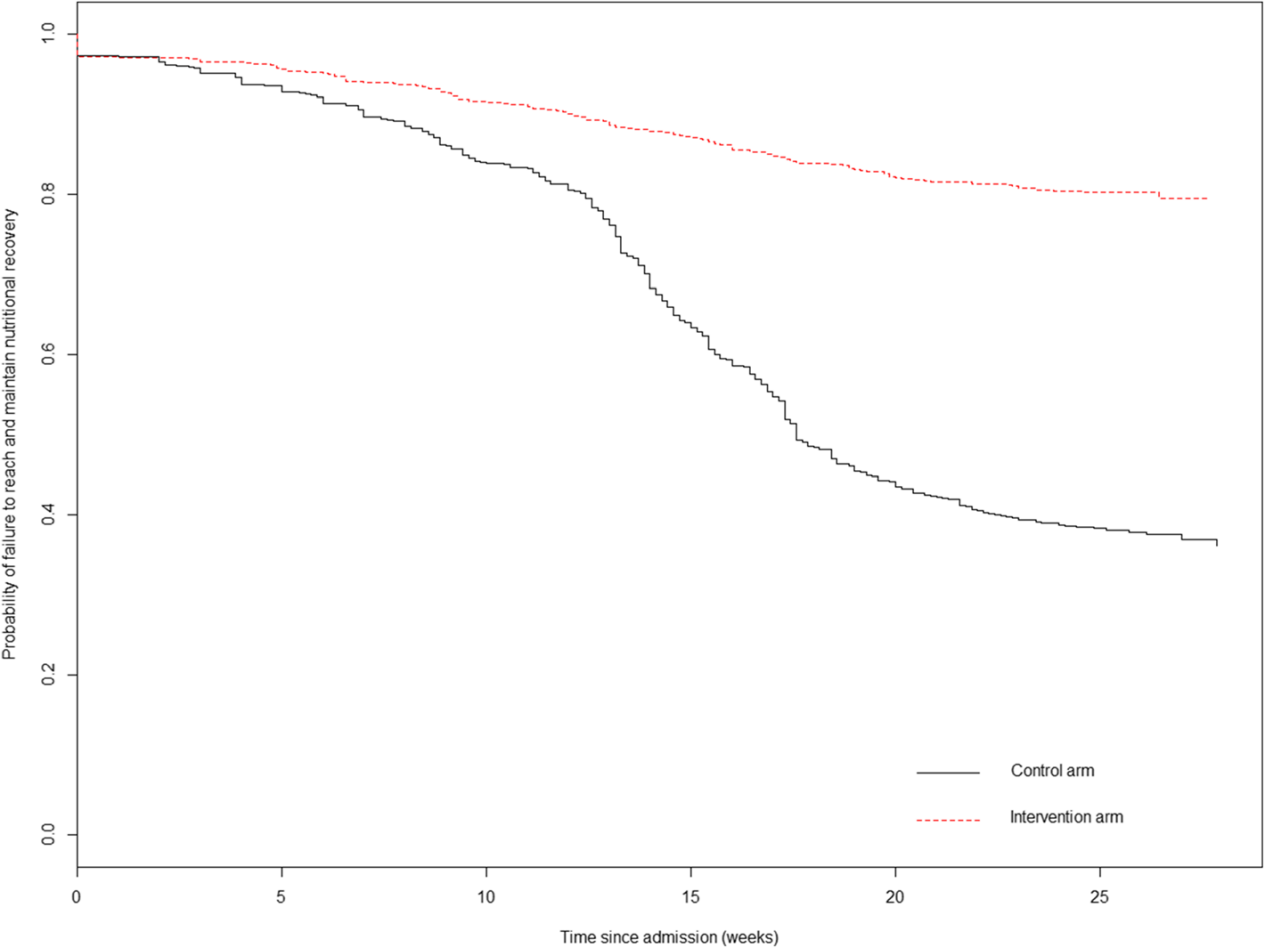
Probability of failure to achieve and maintain nutritional recovery during treatment and follow-up to 6 months from enrolment in the two study groups. Failure of nutritional recovery was defined as dead, non-response to treatment after 12 weeks, a defaulter, a relapse to either SAM or MAM or unknown at 6 months from enrolment. Children were right censored where their nutritional recovery was defined as a WHZ ≥ −1.5 (WHO Growth Standards 2006) or MUAC ≥125 mm and absence of bilateral oedema and they did not relapse. Survival curves of the two groups were compared using the Cox regression analyses with robust estimates of the variance to account for clustering at the health centre level, and *P* value was performed with the robust score test (HR = 0.24, 95% CI 015 to 0.39; *P* < 0.001)

Thus, the cash intervention had a positive effect on all aspects of the management of these children. There was less defaulting, non-response to treatment, withdrawal, and relapse; faster gain in weight and MUAC, particularly after discharge; and actual catch-up in anthropometry relative to WHO standards. The cumulative effect of these factors on the final success or failure of the two different arms of the program to affect the children’s overall recovery up to 6 months is shown in the Kaplan-Meier plot (Fig. 2). It is clear that the proportion of ‛failures’ starts to increase shortly after admission and steadily increases so that by 12 weeks (the maximum time of treatment before declaring the child a non-responder) there was a substantial difference between the groups. There followed a steep decline in the control group as children relapsed without the post-treatment support given to the intervention group. In contrast, the decline in the cash-intervention group was relatively small and steady without any inflection at the time of discharge. Although by the time of discharge the success of the program was above Sphere standards for both groups, albeit much better in the cash group, the overall outcome was dramatically different [43]. After 6 months, 80.4% of the children in the intervention group not only recovered but remained so and on average had significant catch-up in weight and MUAC compared to WHO standards. In contrast, without the cash intervention only 38.8% of the children had an entirely successful outcome.

As the baseline characteristics or health centre performance did not account for the differences, the question arises of whether the difference is due to the way in which the cash was used by the family, and whether it was used specifically to support the child’s recovery or whether it was used by the adults for other purposes. The heads of household reported that they spent 67.2% of the money received to purchase food; 14.8% was dedicated to income-generating activities, followed by clothing (4.3%), water (2.8%), bill payment (2.3%), tuition fees (1.8%), health costs (1.5%) and the remaining 5.3% on other activities and basic needs. The extra expenditure on food should translate to an improvement of the quality of the diet for the index child and the whole family.

Table 6 shows the IDDS, HDDS and FCS at the end of the study and the increments from the baseline values. There was a significant increase in each of these indices of food quality during the course of the study in both groups (*P* < 0.001). However, the increment in the cash-intervention group was very much greater than that in the control group; the increment amounted to between 2.6 times for the index child’s dietary diversity to 5.3 times the control group value for the household diet diversity score. By the end of the study, 60% of the intervention group’s households had achieved a high dietary diversity, whereas only 17% of the non-intervened group had an acceptable dietary diversity at this time. Their food consumption scores mirrored these data with only 3.8% of the group receiving cash having a poor or borderline score compared to about 25% of the control group.

The comparison of the children who relapsed after recovery with those who maintained their recovery is given in Additional files 1 and 2: Tables S1 and S2. The children who relapsed all had highly significant losses of WAZ, WHZ, BMIZ, MUAC-age and MUAC-ht. The increments in diet diversity at both an individual and household level were significantly greater than zero, but very much less than those for the control group (*P* < 0.001).

## Discussion

To our knowledge, this is the only study to assess the direct effect of supporting households with cash during the course of treatment and follow-up of children with SAM. The analysis by intention to treat showed that the cash supplement significantly improved all aspects of treatment. Six months after admission, 80% of the children whose families were given additional support remained within the normal range of WHZ and MUAC. In contrast, less than 40% of those whose families did not receive this additional support had a good outcome; this is not only statistically significant but also biologically highly significant.

For a child to develop severe acute malnutrition shows the child, and presumably the siblings and whole household, to be at particular risk of death and the other serious consequences of being severely malnourished. Follow-up of young children in a DRC community with good medical facilities, but without specific management of SAM, shows that about 5% with a WHZ < –3Z are dead within 3 months; this increases to 15 to 20% at < –4Z and 30% for those approaching –5Z [44–46].

The parents of malnourished children need to choose between attending the health centre and all their other competing activities essential to the integrity of the household. The poorer the household, the more important each individual economic activity is, because the survival of the household is fragile and continuously at risk from even minor additional stress. If the parents consider that the treatment at the health centre is not helping, competing activities critical or the costs of attending excessive, attending will not be a priority and they will default. Thus, the defaulting rate is one measure of the quality of the service provided. Children are much less likely to recover if they default from treatment [47]. The cash incentive could have altered the parents’ perception and priorities so that there were far fewer defaulters in the intervention group from the early stages of treatment. The financial incentive to remain in the program is an important aspect of a CTP.

During treatment the intervention group gained weight faster than the control group, more children recovered and fewer failed to respond to treatment. This occurred despite the same amount of RUTF being dispensed to the families and all other aspects of treatment being the same apart from the cash delivery. The question arises of how the cash delivered to the family effected this improvement in recovery from wasting. We hypothesise that it was due either to the children receiving a higher proportion of the dispensed therapeutic food or to the child getting a higher quality diet from the family pot or a combination of these factors. As most of the funds were spent on food, it is unlikely that the immediate environment (water and sanitation for example) improved over a short time or that there was significantly more health-seeking behaviour.

We collected data on the sharing of the RUTF. The data is not presented because we judged it to be very inaccurate. The respondents from both groups reported very low levels of sharing within the family. In fact, compliance is usually poor in resource-poor settings with extensive sharing and there is an incentive for the families to exaggerate the amount taken by the child in order to ensure continued enrolment in the program [48, 49]. The mean energy cost of tissue deposition when a complete diet is given is about 5 kcal/g of new tissue [50], and the requirement for maintaining body weight is about 100 kcal/kg/day. Thus, as the rates of weight gain were 3.8 to 4.4 g/kg/day, the intakes of these children would average around 120 kcal/kg/day. Thus, the *maximum* average intake of the RUTF is around 70% of the amount dispensed; the actual intake is likely to be much lower because some of the 120 kcal/kg taken by the child will come from the family food and not from the RUTF. Furthermore, if the RUTF were being taken exclusively, there would have been no other foods taken and the IDDS would have shown no diversity at all. As this was not the case, it confirms that the children were indeed consuming less than the computed 70% of the dispensed RUTF. Unrealised low compliance can be a major reason why randomised controlled trials report false negative results [51]. It is possible that in our trial, the extra cash, mostly spent on food, may have resulted in less sharing of the RUTF, because other family members now had adequate wholesome food, but this is unlikely. It is possible that the responses given to the investigators who reported less sharing in other studies were biased. However, the extra cash allowed the intervention households to purchase more expensive nutrient-rich food items, as shown by the increments in the food consumption and diet diversity scores. This, combined with the IYCF counseling, may have resulted in the whole family, including the index child, having a much higher quality family pot to augment the RUTF.

Dietary diversity is associated with a child’s nutritional status [52–54]. Globally less than one third of 6 to 23 month old children meet the minimum criteria for dietary diversity, and only half receive the minimum number of meals required to maintain health [54]. Dietary monotony has been described as the hallmark of poverty and poor nutrition, and indeed, typical child diets in communities and households with high rates of malnutrition are restricted, nutrient-deficient, monotonous and bulky [55]. It is not possible to link poor growth to specific nutrient deficiencies in epidemiological studies because multiple nutrients are required for growth, and deficient diets usually lack several nutrients essential for growth.

In agreement with our results, CTPs in African countries and programs have reported an increase in household consumption with the majority of the additional income from the cash intervention being spent on a variety of foods with a resulting improvement of diet diversity [18–21]. Cash transfers used in social protection programs are different from cash transfers in an emergency. In our case, the transfer took place during a relatively short period, when vulnerability was particularly high and the targeting criteria were clear; this may have affected how the money was used, because it was known by the beneficiaries that the cash income would not be sustained. The decision to use unconditional instead of conditional cash transfer in our study was based on the market analysis and KAP survey. Any consequent change in diet quality will depend upon the type and cost of the foods that are available for purchase locally, which will vary from place to place and from season to season. The children’s intake will also depend on how the households appreciate the foods that are usually more expensive than the family staple and how they apportion these foods between family members — in particular to the index child. The IDDS scores indicate that the individual children’s diet was greatly improved in the intervention group.

The most dramatic finding of our study was the difference in the relapse rate between the children of households who received ongoing support to the end of the 6-month period and those who were simply returned ‛cured’ to their households. Relapse after discharge was the main reason for failure of the program without the cash transfer; of those admitted initially less than 40% were deemed a success after 6 months. These are the most vulnerable children in the community; after treatment they are returned to exactly the same environment and circumstances that they endured whilst becoming malnourished. Although after treatment they are older and healthier and thus more likely to demand and receive their share of the household food, they are still at high risk of relapse without continued support.

The relapse rate without family support after discharge varies greatly from study to study [47], but is often very high. Examples of follow-up studies include two from Malawi, one where moderately malnourished children were followed for 12 months, only 63% remained well nourished, 17% relapsed to MAM and 10% developed SAM, 4% died and 7% were lost to follow-up [56]; in another 25% of the children died after discharge [57], although many of these children had HIV. In one study in Bangladesh, 21% had either died or relapsed to SAM [58] after discharge, but in another study less than 1% relapsed although 2.3% died and 7.5% were lost to follow-up [59]. Little has changed since the early studies, as 8.9% of children were dead within 3 months of discharge having been treated for SAM in a recent study from Dhaka [60]. In Kenya, 1.5 years after discharge, 36% were dead and 28% were again underweight [61]. In Burkina Faso, after 1.1 years the relapse rate was 15.5% with 2.2% of children dying; in this study the loss to follow-up was very high at 34% [62]. In all such studies the relapse and death rates after discharge are minimum values, because a higher proportion of the ‛lost to follow-up’ children are likely to be lost because of death.

The state of food security at discharge has a decisive effect in countries with seasonality. This is elegantly shown in a study from India. Discharge when food security is low was followed by a relapse rate of 35% to MAM and 6.5% SAM; with moderate food security this fell to 29% and 3.8% and with high food security to 8.7% and 0.7% respectively [31]. These marked differences in relapse rate following discharge show the critical effect food security has on the subsequent fate of the children treated for SAM.

Thus, the very large differences between the relapse rates during food-insecure and food-secure times is artificially mirrored in the DRC with our study. The cash converted the food-insecure households with restricted diets and high levels of malnutrition to relatively food-*secure* households in the same area at the same time as the control group households remained food-insecure. The difference in outcome for the two groups is clear. The intervention children continued to catch up from –1.5Z weight-for-height and MUAC for age towards the median; in contrast, the control group without this household support significantly deteriorated with a high proportion of the children relapsing.

Other forms of post-discharge support may also have a beneficial impact on the further fate of the children; for example, a quasi-controlled analysis showed a better outcome of MAM with prolonged supplementary feeding [63]. Cash may not be sufficient to give the best post-discharge outcome for the children; a preventive study in Niger showed that cash plus highly nutritious supplements was superior to cash alone [64], although in our study there did not appear to be significant anthropometric relapse (1.1%) in the intervened group given cash alone post-discharge. There are *inter alia* differences in the types of food available, cultural practices, taboos, woman’s roles, seasonality and climate between the DRC and Niger. This raises the question of the external validity of such studies. However, it is noteworthy that IYCF was not included in the Niger study, and it is possible that such counselling affected the choices, behaviour and disbursements of the recipient households in our study to the benefit of the children.

There was no difference by group in the proportion of children developing oedema after discharge. We do not have an explanation for this observation, but it may depend upon individual nutrients, such as sulphur or particular antioxidants, being generally deficient from the foods available in this area [65].

Our study emphasises that the protocols specifically developed for short-term relief in emergency situations may not be sufficient for use in impoverished communities in a developmental context. Having identified households with a malnourished child in the poorer sections of the society, giving short-term treatment to increase the weight of the child is appropriate to prevent imminent death, but is insufficient when the ‛cured’ children are simply returned to their original poverty-stricken households without other interventions. In this context the ‛emergency’ home treatment should be combined with ‛developmental’ support to the family that can be sustained in the longer term and lead to an improvement in their circumstances. It is unknown what happened to the children in this study when the cash transfer program ceased, because the children were not followed for logistical reasons. They may have deteriorated subsequently in the same way that the control group deteriorated without family support. Given the dramatic findings of this study, in terms of relapse, longer term follow-up should be investigated in further studies.

Although the increases in food diversity and food consumption scores were much greater in the intervention group, there was also an improvement in the control group which was significantly greater than their baseline assessments. This could be due to temporal changes in the whole community; alternatively, it could be ascribed to the IYCF counselling. Whatever the cause, it is clear that IYCF counselling did not have a dramatic effect on preventing deterioration or relapse in the control group. Knowledge about IYCF by the household could not compensate for the effect of poverty on their ability to purchase higher quality foods and presumably to follow the advice given about young child feeding. However, IYCF was probably critical for the changes in the intervention group, who now had the resources to implement the advice given during counselling.

It is important that distribution of cash respects the autonomy of households to decide how to best meet their own requirements. However, we expect that any significant effect on child nutritional status depends on the duration of exposure to the intervention and the amount of cash transfer received per household, per month, relative to the local costs and the local availability of high-quality food. Information on program costs, although a key indicator for public health decision-makers and program managers, was not an objective of this study. Nevertheless, it usually costs less to get cash transfers to people than in-kind assistance because aid agencies do not need to purchase, transport, store and then pay to distribute relief goods. A four-country study comparing cash transfers and food aid found that 18% more people could be assisted at no extra cost if everyone received cash instead of basic food (i.e. not including fresh foods) [66]. In Ethiopia, the World Food Programme found cash to be more cost efficient than food aid by 25–30% [9]. Furthermore, when delivering assistance in conflict zones, especially food aid, there are additional difficulties. For example, in Somalia, only 35% of food aid budgets went to beneficiaries, compared to 85% of cash transfer budgets [67].

In the DRC, and other large countries with a relatively scattered population and poor transport, preventive programs like general food distribution or blanket feeding are logistically too difficult and expensive and so have never been implemented country-wide. Supplementary feeding programs for treating MAM are sporadic and not functional in most of the DRC. In this context, CTPs should be considered as an alternative to in-kind assistance and services or as a complement to more traditional interventions. Normally national protocols for treating SAM state that children who have recovered from SAM should be enrolled in a supplementary feeding program to be followed and receive a fortified food ration for at least 3 months after discharge. This provision is frequently unrealistic. The results of the present study, which shows that a cash supplement effectively prevents relapse and allows for continued catch-up, demonstrate CTP to be a viable and more easily implemented alternative to a supplementary feeding program [68, 69]. Furthermore, if the cash is used to generate an ongoing income through minor entrepreneurial activities or other capital expenditures, then these poorest of families and their at-risk children may be helped in the longer term. The emergency non-governmental organisations (NGOs) which spearheaded the community management of SAM should consider adopting a developmental approach after treatment of the children with SAM, to alleviate poverty for such families rather than simply dispensing food and antibiotics.

Most programs for managing severely malnourished children do not automatically include a follow-up program and only report the (usually) excellent ‛recovery’ rates. Such would have been the results of both arms of our study. By including the follow-up in the assessment of our program, we have shown that many reports can be misleading in terms of the overall success of a program. We strongly recommend that such continued support and its evaluation should be routine for all CMAM/IMAM programs in relatively stable countries.

This study showed that there was no catch-up in height-for-age in either group. Thus, the program had no effect on stunting. It may be that increased rates of normal growth, indicated by a height increase, are delayed beyond the study period, as observed by Heikens et al. [70]. Nevertheless, in none of the follow-up studies quoted above has a significant height increment been described. Studies of children involved in programs to prevent malnutrition have shown that the intervention prevents deterioration in height-for-age among the beneficiaries [71] but without evidence of catch-up. Nevertheless, catch-up has been shown, only after full recovery in weight-for-height, when high-quality diets are given under supervision [72]. A change in home environment has frequently led to catch-up in height [73], but rarely if the home environment does not change [74]. Thus, although our program was very successful in correcting wasting and preventing relapse, there was neither a significant effect on stunting nor on the recurrence of oedematous malnutrition. The nutritional and other requirements to correct these deficits appear not to depend upon or be prevented by the interventions tested in this study.

### Limitations of the study

It was not possible, of course, to blind the participants to the transfer of cash into their hands. Indeed, this intervention might have been the incentive to continue participation in the study and may have affected the results by preventing defaulting which was then analysed by intention to treat so that all defaulting children were included in the analyses and, where possible, followed up with home visits. Although the health centre staff were not involved in any way with the cash transfer, it is difficult on a practical level to prevent the service personnel from becoming aware of who received the intervention and who did not.

As the participants were not blinded, it is possible that the intervention group’s respondents were more disposed towards the study than those of the control group, which could have affected the answers they gave to the questionnaires. We do not think that this is likely to have biased the results, as they both reported clearly erroneous reports of RUTF sharing to the same extent, and there were similarities of the two groups’ responses at baseline. Nevertheless, it was not possible to verify the accuracy of the other questionnaire data with direct home observations. Clearly, this potential bias will not affect the anthropometric data.

There is likely to have been an ascertainment bias so that the patients were not properly representative of severe malnutrition in the community, because those who were recruited by MUAC criteria were selected from community screening and those who were selected by WHZ criteria were taken from attendees at the health centre. Survey data from the DRC indicate that 32% of the severely malnourished children by WHZ have a MUAC above the cut-off point for SAM [75]. They will be under-represented in the patient population. If the WHZ children are more severely ill than those identified by MUAC, it could account for the fact that none of the intervention children relapsed using MUAC criteria, but 1.1% relapsed using WHZ criteria. However, this difference is quite trivial compared to the difference in the two arms of the study, so we do not consider this to have resulted in a major bias.

As there is no real seasonality in this location and as the two arms were conducted concurrently, we do not consider the time of discharge as a cause of bias. The data for the non-intervened group are consistent with the reports of other programs of outpatient treatment in many other locations, and their rates of relapse are similar to those of many other reports. We therefore consider the study to have reasonable external validity for the control group. The effect of the intervention, however, is dependent upon variables such as the availability of varied nutrient-dense products on the market, reasonable market access for the participants and reasonable price stability; therefore, the external validity of the intervention arm needs to be confirmed in other contexts with differing potential effectors.

This research demonstrates the benefits of cash assistance; any potential negative impacts were not considered or examined (such as the extra cash in the society increasing market prices to the detriment of the control group). However, one possible negative effect of a therapeutic treatment program is that the child will be purposefully kept malnourished in order to receive the benefit. This is not thought to be a problem in the present study, as it was made clear to the beneficiaries of the intervention arm that they would receive the cash transfer monthly for 6 months independently of the recovery rate of the child, provided that they did not default from the program. Thus, the cash is likely to have deterred defaulting (a good outcome), but there was no incentive to maintain the child in a malnourished state to continue to benefit from the program. It is possible that such an effect was present for some children in the control arm of the study; however, the mean length of stay under treatment was not different between the groups. If this did occur, it is a further benefit of the cash transfer for the intervention group.

The study does not provide evidence of a greater positive effect of providing cash assistance rather than in-kind or other forms of assistance. The study estimates the impacts of cash when US$240 was delivered per household over the course of 6 months to children with SAM. The findings should not be extrapolated to different amounts or time frames. Moreover, this trial was conducted in a semi-urban area of the DRC without marked seasonal variation that had during the study a relatively poor food insecurity and a high prevalence of wasting. Therefore, our results need to be extrapolated with care and interpreted within the given context.

## Conclusions

This study shows that giving cash in impoverished communities can be effective in improving the outcome of children treated for SAM and provides a safety net that prevents relapse and allows for continued catch-up in weight and MUAC up to 6 months from admission. This very positive impact over a relatively short time on the children’s nutritional status is most easily explained by the improved access to high-quality food, enabling households not only to meet minimal needs for survival but also to diversify their diet within a society characterised by a high level of endemic malnutrition. In the DRC, where supplementary feeding interventions are logistically difficult and expensive to implement, to reduce acute malnutrition and improve its coverage, carefully designed cash transfer is shown to be a viable and highly effective intervention. Such innovative programs merit further investigation in different contexts to assess their cost-effectiveness compared to other interventions. Nutritional rehabilitation programs, often using the procedures derived directly from emergency relief operations, should always consider the feasibility of incorporating developmental programs such as micro-credit, home gardening, etc. into their procedures to enable the gains from acute nutritional intervention to be sustained.

## Additional files

Additional file 1: Table S1. Changes in diet diversity and food consumption score between children who relapsed and recovered. (DOC 35 kb)

Additional file 2: Table S2. Changes in anthropometric indicators between children who relapsed and those who did not relapse after discharge from therapeutic home treatment for severe acute malnutrition. (DOC 51 kb)

## Abbreviations

BMIZ: Body mass index for age Z-score
CI: Confidence interval
CMAM: Community-based management of acute malnutrition
CTP: Cash transfer program
DRC: Democratic Republic of the Congo
FCS: Food Consumption Score
HAZ: Height-for-age Z-score
HDDS: Household Dietary Diversity Score
HEA: Household Economy Approach
HR: Hazard ratio
IDDS: Individual Dietary Diversity Score
IMAM: Integrated management of acute malnutrition
ITP: Inpatient therapeutic program
IYCF: Infant and young child feeding
KAP: Knowledge, Attitude and Practices survey
MAM: Moderate acute malnutrition
MUAC: Mid-upper arm circumference
MUACZ-age: Mid-upper arm circumference for age Z-score
MUACZ-ht: Mid-upper arm circumference for height Z-score
NGO: Non-governmental organisation
OTP: Outpatient therapeutic program
RUTF: Ready-to-use therapeutic foods
SAM: Severe acute malnutrition
WAZ: Weight-for-age Z-score
WHZ: Weight-for-height /length Z-score
