# Supplementary material for: Effects of unconditional cash transfers on the outcome of treatment for severe acute malnutrition (SAM): a cluster-randomised trial in the Democratic Republic of the Congo
Source: BMC Med. 2017 Apr 26;15:87. doi: 10.1186/s12916-017-0848-y (PMC5405483; doi:10.1186/s12916-017-0848-y)
Supplement: Supplementary file 1 — Changes in diet diversity and food consumption score between children who relapsed and recovered. (DOC 35 kb) [file 12916_2017_848_MOESM1_ESM.doc]

**Additional file 1: Table S1:** Changes in diet diversity and food consumption score between children who relapsed and recovered.

| **Parameters** | **Relapse** | | **Recovery** | | **mean of the differences** | | **P value^1^** |
| --- | --- | --- | --- | --- | --- | --- | --- |
|  | **mean** | **SD** | **mean** | **SD** | **mean** | **(95 % CI)** |  |
| Relapsed to MAM (n=383) | | | | | | | |
| IDDS change | 0.62 | ± 1.29 | 1.34 | ± 1.44 | 0.72 | (0.55 to 0.88) | <0.001 |
| HDDS change | 0.82 | ± 1.84 | 1.80 | ± 1.95 | 0.98 | (0.75 to 1.22) | <0.001 |
| FCS change | 8.81 | ± 15.00 | 15.55 | ± 14.50 | 6.74 | (4.89 to 8.58) | <0.001 |
| Relapsed to SAM (n=97) | | | | | | | |
| IDDS change | 0.82 | ± 1.38 | 1.35 | ± 1.44 | 0.53 | (0.22 to 0.82) | <0.001 |
| HDDS change | 0.71 | ± 1.42 | 1.80 | ± 1.95 | 1.09 | (0.77 to 1.41) | <0.001 |
| FCS change | 9.42 | ± 14.15 | 15.60 | ± 14.52 | 6.18 | (3.12 to 9.24) | <0.001 |

^1^ Change within groups assessed using unpaired Student’s t tests.
