# Supplementary material for: Effects of unconditional cash transfers on the outcome of treatment for severe acute malnutrition (SAM): a cluster-randomised trial in the Democratic Republic of the Congo
Source: BMC Med. 2017 Apr 26;15:87. doi: 10.1186/s12916-017-0848-y (PMC5405483; doi:10.1186/s12916-017-0848-y)
Supplement: Supplementary file 2 — Changes in anthropometric indicators between children who relapsed and those who did not relapse after discharge from therapeutic home treatment for severe acute malnutrition. (DOC 51 kb) [file 12916_2017_848_MOESM2_ESM.doc]

**Additional file 2: Table S2:** Changes in anthropometrics indicators between children who relapsed and those that did not relapse after discharge from therapeutic home treatment for severe acute malnutrition.

| **Parameters** | **Relapse** | | **No-relapse** | | **mean of the differences** | | **P value^1^** |
| --- | --- | --- | --- | --- | --- | --- | --- |
|  | **mean** | **SD** | **mean** | **SD** | **mean** | **(95 % CI)** |  |
| Relapsed to MAM (n=383)^2^ |  |  |  |  |  |  |  |
| HAZ change (Zscore/month), mean (SD) | 0.003 | 0.157 | -0.014 | 0.173 | -0.017 | (-0.037 to 0.002) | 0.093 |
| Weight change (gram/kg/day), mean (SD)^4^ | 0.265 | 0.588 | 0.896 | 0.670 | 0.631 | (0.554 to 0.707) | <0.001 |
| WAZ change (Zscore/month), mean (SD)^4^ | -0.058 | 0.126 | 0.086 | 0.142 | 0.144 | (0.128 to 0.160) | <0.001 |
| WHZ change (Zscore/month), mean (SD)^4^ | -0.099 | 0.188 | 0.142 | 0.213 | 0.241 | (0.216 to 0.266) | <0.001 |
| BMIZ change (Zscore/month), mean (SD)^4^ | -0.088 | 0.213 | 0.149 | 0.249 | 0.237 | (0.209 to 0.265) | <0.001 |
| MUAC change (mm/day), mean (SD) | -0.001 | 0.039 | 0.057 | 0.049 | 0.058 | (0.052 to 0.063) | <0.001 |
| MUACZ-age change (Zscore/month), mean (SD) | -0.038 | 0.118 | 0.129 | 0.137 | 0.167 | (0.151 to 0.182) | <0.001 |
| MUACZ-ht change (Zscore/month), mean (SD) | -0.035 | 0.120 | 0.137 | 0.141 | 0.172 | (0.156 to 0.187) | <0.001 |
| Relapsed to SAM (n=97)^3^ |  |  |  |  |  |  |  |
| HAZ change (Zscore/month), mean (SD) | -0.001 | 0.191 | -0.014 | 0.173 | -0.013 | (-0.05 to 0.027) | 0.500 |
| Weight change (gram/kg/day), mean (SD)^4^ | 0.078 | 0.701 | 0.896 | 0.670 | 0.819 | (0.622 to 1.015) | <0.001 |
| WAZ change (Zscore/month), mean (SD)^4^ | -0.104 | 0.167 | 0.086 | 0.142 | 0.190 | (0.144 to 0.237) | <0.001 |
| WHZ change (Zscore/month), mean (SD)^4^ | -0.204 | 0.253 | 0.142 | 0.213 | 0.346 | (0.275 to 0.417) | <0.001 |
| BMIZ change (Zscore/month), mean (SD)^4^ | -0.203 | 0.282 | 0.149 | 0.249 | 0.352 | (0.274 to 0.432) | <0.001 |
| MUAC change (mm/day), mean (SD) | 0.005 | 0.057 | 0.057 | 0.049 | 0.052 | (0.040 to 0.064) | <0.001 |
| MUACZ-age change (Zscore/month), mean (SD) | -0.025 | 0.172 | 0.129 | 0.138 | 0.154 | (0.117 to 0.191) | <0.001 |
| MUACZ-ht change (Zscore/month), mean (SD) | -0.008 | 0.174 | 0.137 | 0.142 | 0.145 | (0.136 to 0.183) | <0.001 |

^1^ Change within groups assessed using unpaired Student’s t tests.

^2^ Relapse to MAM was defined as the development of a WHZ <-2.0 and ≥-3.0 (WHO Growth Standards 2006) or MUAC<125mm and ≥115mm (without bilateral oedema) at least once during the monthly follow-up visits; without the child developing SAM criteria during any other follow-up visit.

^3^ Relapse to SAM was defined as development of a WHZ <−3.0 (WHO Growth Standards 2006) or MUAC <115mm or presence bilateral oedema at least once during the monthly follow-up visits.

^4^ Children with oedema were excluded from the analysis of parameters including weight.
